# Iron, Oxidative Damage and Ferroptosis in Rhabdomyosarcoma

**DOI:** 10.3390/ijms18081718

**Published:** 2017-08-07

**Authors:** Alessandro Fanzani, Maura Poli

**Affiliations:** Department of Molecular and Translational Medicine (DMMT), University of Brescia, Viale Europa 11, 25123 Brescia, Italy

**Keywords:** iron, ferroptosis, oxidative damage, rhabdomyosarcoma

## Abstract

Recent data have indicated a fundamental role of iron in mediating a non-apoptotic and non-necrotic oxidative form of programmed cell death termed ferroptosis that requires abundant cytosolic free labile iron to promote membrane lipid peroxidation. Different scavenger molecules and detoxifying enzymes, such as glutathione (GSH) and glutathione peroxidase 4 (GPX4), have been shown to overwhelm or exacerbate ferroptosis depending on their expression magnitude. Ferroptosis is emerging as a potential weapon against tumor growth since it has been shown to potentiate cell death in some malignancies. However, this mechanism has been poorly studied in Rhabdomyosarcoma (RMS), a myogenic tumor affecting childhood and adolescence. One of the main drivers of RMS genesis is the Retrovirus Associated DNA Sequences/Extracellular signal Regulated Kinases (RAS/ERK)signaling pathway, the deliberate activation of which correlates with tumor aggressiveness and oxidative stress levels. Since recent studies have indicated that treatment with oxidative inducers can significantly halt RMS tumor progression, in this review we covered different aspects, ranging from iron metabolism in carcinogenesis and tumor growth, to mechanisms of iron-mediated cell death, to highlight the potential role of ferroptosis in counteracting RMS growth.

## 1. Introduction

Iron is the most abundant heavy metal in mammals (about 3–5 g in human adults), as it is involved in a number of biological processes, ranging from metabolism and oxygen transport to DNA synthesis and antioxidant defense. Many redox enzymes involved in cellular respiration use iron-sulfur (Fe-S) clusters as preferred cofactors, named ferredoxins, such as nicotinamide adenine dinucleotide (NADH) dehydrogenase, hydrogenases, coenzyme Q—cytochrome c reductase, succinate-coenzyme Q reductase and other components of the mitochondrial electron transport chain. In addition, heme iron is used for oxygen transport by hemoglobin and myoglobin and for the detoxification of reactive oxygen species (ROS) by catalase and superoxide dismutase enzymes. Often tumor cells show a marked alteration in metabolism leading to intracellular accumulation of iron, which is strongly utilized for tumor growth and angiogenesis [1]. Accordingly, some anti-tumor strategies using metal chelators have been successfully developed [2]; however, iron deprivation is potentially harmful to non-tumor cells, as it may favor cell death by apoptosis [3,4,5]. Recently, an iron-dependent type of programmed cell death has been identified, named ferroptosis [6,7,8]. High intracellular iron concentrations can trigger ferroptosis by enhancing the generation of lipid peroxides, and this can be reverted using iron chelators [9]. Currently, the use of ferroptosis as a weapon against tumors is of increasing interest, as tumor cells traditionally exhibit high endogenous oxidative stress levels due to gene aberrations promoting continuous cell cycles (gain of RAS, and myelocytomatosis viral related oncogene MYC) and resistance to cell senescence (P53 loss) [10]. In the next paragraphs we will describe the complex role of iron in cancer and discuss the available evidence on iron and ferroptosis in rhabdomyosarcoma (RMS), the most frequent soft tissue tumor affecting patients of pediatric and adolescent age.

## 2. The Pleiotropic Role of Iron in Cancer

**Figure 1:**
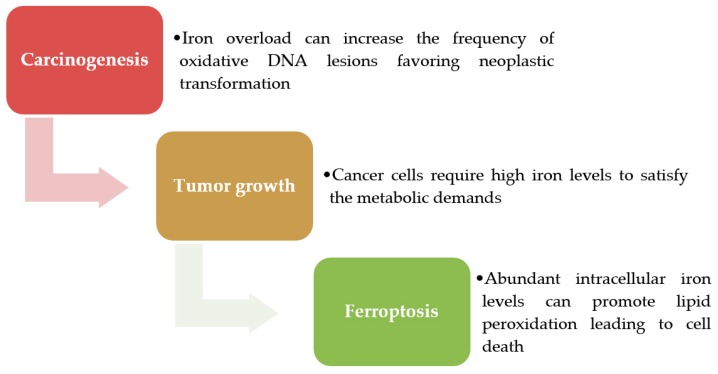
Iron in cancer. Iron can promote carcinogenesis by oxidative stress that increases DNA damage. Following neoplastic transformation, tumors utilize various mechanisms to maintain the high intracellular iron free levels necessary for tumor growth. Over time the iron overload could become deleterious by inducing lipid peroxidation and ferroptosis.

Deregulation of iron homeostasis may have a different impact in cancer depending on the stage of tumor progression, as summarized in Figure 1. As detailed below, iron levels influence carcinogenesis, tumor progression and sensitivity to ferroptosis.

### 2.1. Iron-Induced Oxidative Stress Plays a Role in Carcinogenesis

Iron overload has been associated with a higher risk of carcinogenesis, as observed in some pathological conditions, including hereditary hemochromatosis, ovarian endometriosis, chronic inflammation induced by viral hepatitis B/C, and exposure to foreign asbestos nanoparticles [11,12]. This is due to the ability of iron to promote DNA damage, as described in a model of renal carcinogenesis [13]. In particular, the most frequent DNA damage is the 8-oxo-7,8-dihydro-2′-deoxyguanosine (8-oxo-dG), resulting from the oxidation of guanine, which potently induces G:C→T:A transversion mutations. Several enzymes involved in DNA repair have been identified, such as the 8-oxoguanine DNA glycosylase 1 (OGG1) [14,15] and a homologue MutT variant first isolated in a mutant strain of *E. coli* [16]. Interestingly, patients with chronic hepatitis C, who have abnormally high levels of 8-oxo-dG and repair enzymes, are protected from the formation of pre-neoplastic lesions and hepatocellular carcinoma by phlebotomy and a low iron diet [17,18]. Other studies further confirmed that phlebotomy twice a year for five years significantly protects against cancer events [19]. Altogether these data indicate that iron-induced oxidative stress can represent a critical factor for carcinogenesis induced by DNA mutagenesis [20,21,22].

### 2.2. Iron Addiction Is a Hallmark of Cancer Cells

**Figure 2:**
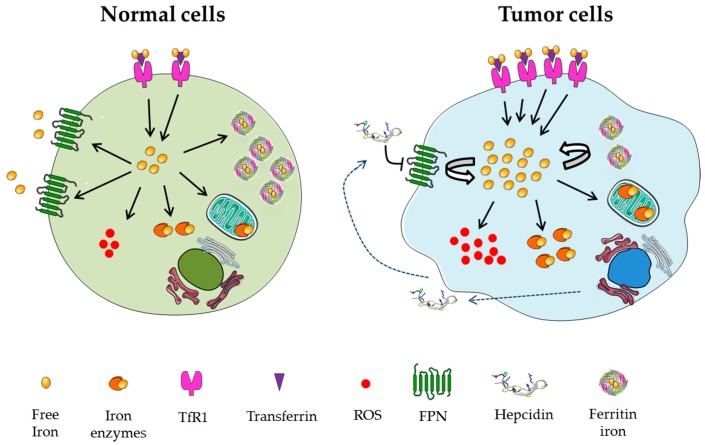
Iron addiction of tumor cells. In normal cells the transferrin receptor 1 (TfR1)-mediated iron absorption is counter balanced by iron efflux via ferroportin (FPN); the free iron pool is used by cytosolic and mitochondrial enzymes and the excess is stored by ferritins to prevent cytotoxicity. As a result, only a minor part of the intracellular iron, present as a free labile pool, can stimulate the formation of Reactive Oxygen Species ROS. In contrast, tumor cells often show higher levels of TfR1, down-regulation of FPN mediated by secreted hepcidin and lower levels of ferritins, which leads to an increased intracellular labile iron pool; despite this it’s mostly being utilized for tumor growth by cytosolic and mitochondrial iron enzymes, the exceeding amount can promote increased oxidative stress via ROS accumulation. The figure was adapted using a template on the servier medical art website (available online: www.servier.com) licensed under the creative commons attribution 3.0 unported license (available online: http://creativecommons.org/license/by/3.0/).

In humans, iron homeostasis is under the control of mechanisms that coordinate the absorption, export, storage, transport and utilization of iron. The amount of iron circulating in serum and available to tissue may originate from the diet (about 1–2 mg/day), the recycling of hemoglobin by macrophages (about 20 mg/day) and hepatic stores (0.5–1 g) [23]. Iron release from these sources is controlled by hepcidin, a circulating 25 amino acid peptide hormone that reduces systemic iron availability via the binding and degradation of ferroportin (FPN), the only known cellular iron exporter [24]. Dietary iron absorption is mediated by the divalent metal transporter (DMT1) and the duodenal cytochrome b (Dcytb), both iron-regulated [25]. Plasma iron is delivered by transferrin to all tissues presenting the transferrin receptor 1 (TfR1), which mediates its endocytosis [23]. Iron is then reduced and delivered throughout the cytosol to mitochondria for the synthesis of heme groups, Fe/S complexes and iron enzymes, whereas the excess is sequestered and stored by ferritins [26] (Figure 2). The amount of iron bound to ferritins (up to 4500 atoms) can be recycled via a recently identified mechanism mediated by a nuclear receptor coactivator 4 (NCOA4), which targets H-ferritin to lysosomal degradation [27]. As a result of these coordinated events, in non-tumor cells only a minor fraction of free labile iron is present in the cytosol, usually complexed with low molecular weight molecules including glutathione (GSH), citrate, sugars, ascorbate, nucleotides, and also enzymes [23]. On the other hand, an abnormal increase of the intracellular free iron pool is observed in cancer cells, as described in ovarian, breast, lung, prostate, and pancreatic tumors, colorectal hepatoma, gastric and hematological cancers, and melanoma [1]. This effect, commonly referred as to “iron addiction” [1], is the result of the deregulation of different mechanisms. For example, altered MYC expression, which plays a key role in cell transformation, is also responsible for iron metabolism by modulating the activity of the iron responsive protein-2 (IRP2), which in turn orchestrates the expression of different iron proteins [28]. As depicted in Figure 2, cancer cells show increased iron absorption due to high expression of TfR1, a downstream target of MYC oncoprotein [29] and hypoxia inducible factors (HIFs) [30], as observed in breast, renal, and ovarian tumors [31,32,33]. In addition, the down-regulation of FPN mediated by hepcidin can limit iron export [34], whereas MYC and RAS can promote the release of stored iron by the degradation of H-ferritin [35,36]. Finally, the stroma, endothelial and inflammatory cells composing the tumor niche can release iron to feed the neighboring tumor cells through a concerted upregulation of FPN and down-regulation of ferritin and heme-oxygenase [37]. 

### 2.3. Iron as a Trigger of Ferroptosis in Tumor Cells

**Figure 3:**
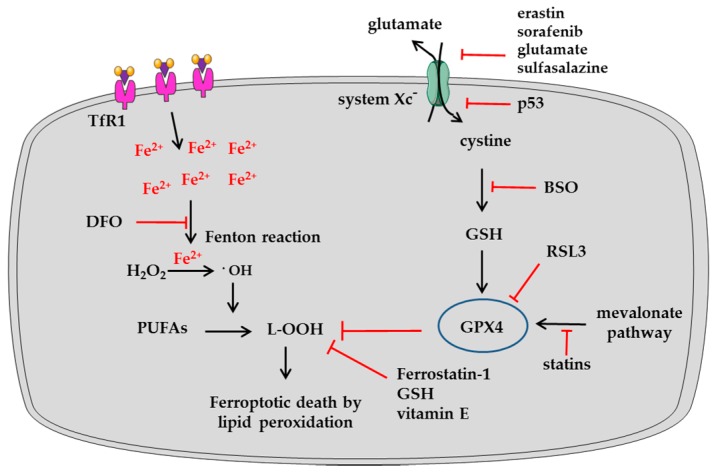
Intracellular levels of iron, glutathione (GSH) and polyunsaturated fatty acids (PUFAs) influence ferroptosis. The abundant intracellular iron through the Fenton reaction can result in higher formation of hydroxyl radicals (^•^OH), the most reactive ROS (Reactive Oxygen Species) intermediates. These promote conversion of PUFAs into lipid hydroperoxides (L–OOH) that lead to ferroptosis. This process can be exacerbated pharmacologically by the inhibition of glutathione peroxidase 4 (GPX4), the enzyme responsible for L–OOH neutralization, by treatment with RAS selective lethal 3 (RSL3). Alternatively, GPX4 activity may be inhibited by a depletion of GSH via treatment with inhibitors of the system Xc^−^ responsible for cystine uptake (such as erastin, sorafenib, glutamate and sulfasalazine) or with buthionine-sulfoximine (BSO), an inhibitor of the first reaction of GSH biosynthesis. The system Xc^−^ is also transcriptionally repressed by p53. In addition, treatment with inhibitors of the mevalonate pathway, such as statins, affect GPX4 synthesis and stimulate ferroptosis. On the other hand, strategies to prevent ferroptosis include treatment with iron chelators such as deferoxamine (DFO) and neutralization of L-OOH by treatment with lipid peroxidation inhibitors (Ferrostatin-1) and antioxidant scavengers (GSH, Vitamin E).

Ferroptosis is an iron-dependent form of programmed cell death [6] that differs from canonical apoptosis, necroptosis or autophagy in its morphological features and biochemical pathways [38,39,40]. As depicted in Figure 3, intracellular iron accumulation yields hydroxyl radicals via the Fenton reaction, therefore promoting the oxidation of polyunsaturated fatty acids (PUFAs, such as linoleic, arachidonic and docosahexaenoic acids). The resulting lipid peroxides and hydroperoxides [41] cause severe structural/functional alterations of cell membranes [8]. Treatments with iron chelators, antioxidant scavengers (like GSH or Vitamin E), and specific inhibitors of lipid peroxidation (like Ferrostatin-1) can prevent ferroptosis activation [6,42]. Moreover, glutathione peroxidase 4 (GPX4), a selenoprotein, protects from ferroptosis [43] as it catalyzes the endogenous neutralization of lipid hydroperoxides (L-OOH) into innocuous lipid alcohols [44,45]. Indeed, the treatment of cells with the GPX4 inhibitor RSL3 (RAS selective lethal 3) rapidly induces ferroptosis [43]. On the other hand, erastin (eradicator of RAS and ST-expressing cells) facilitates ferroptosis preferentially in RAS-positive cancer cells [46,47]. Preliminary studies have shown that erastin inhibits the membrane potential of mitochondria [48], while subsequent studies have elucidated how erastin reduces GPX4 activity [6]. Specifically erastin, similarly to sulfasalazine [6], sorafenib [49,50] and glutamate [6], inhibits cystine absorption from the extracellular space mediated by system Xc^−^, a glutamate-cystine antiporter [6,51]; this reduces the intracellular biosynthesis of GSH, leading to subsequent impairment of GPX4 activity. After its recent discovery in 2012, the importance of ferroptosis has attracted much interest as it represents a potential mechanism for controlling tumor growth. To date, different types of tumor have shown sensitivity to ferroptosis inducers [10], including diffuse large B-cell lymphoma, renal cell carcinoma, liver cancer, cervical carcinoma, osteosarcoma, prostate adenocarcinoma, pancreatic carcinoma, and ovarian carcinoma [43,52,53,54]. In addition, several proteins and pathways have been described as modulators of ferroptosis (Table 1). However, few studies have so far documented the ferroptosis process in sarcomas. 

## 3. Ferroptosis and Rhabdomyosarcoma

### 3.1. Rhabdomyosarcoma Is a Soft Tissue Sarcoma Characterized by Oxidative Stress

Sarcomas are mesenchymal tumors originating from cell precursors committed to form fat, blood vessels, nerves, bones, muscles, deep skin tissues, and cartilage. The latest classification by the World Health Organization [69] divided sarcomas into non-soft tissue sarcomas and soft-tissue sarcomas (STS). The former includes bone sarcomas such as osteosarcoma, Ewing’s sarcoma, and chondrosarcoma; while the latter includes a family of more than 50 neoplasms representing about 20% of childhood and adolescence tumors and 1% of all adult cancers. RMS arises from cell progenitors committed to skeletal muscle [70,71,72,73] and is the most common STS in patients of pediatric and adolescent age [74,75]. RMS subdivides into four main subtypes depending on histology appearance, tumor location, age of onset, and molecular drivers (Table 2). 

The two predominant histotypes are the embryonal (ERMS) and the alveolar (ARMS) forms that commonly affect children under 10 years or adolescents/young adults, respectively [88]. Currently, chemotherapy, radiotherapy, and surgery are used to treat this aggressive tumor, with a five-year survival rate of higher than 70% in patients with localized disease; however, the overall survival of patients with metastasis remains low [89,90]. Different types of molecular drivers have been identified for each RMS subtype (Table 2) [91]. The most aggressive ARMS is dominated by a chromosomal translocation t(2;13)(q35;q14) that juxtaposes the DNA binding domain of the *PAX3* gene in a frame with the activation domain of the *FOXO1* gene, giving rise to a Pax3-Foxo1 chimeric transcription factor that is found in 70% of ARMS cases and is considered a predictor of poor prognosis [92,93]. ERMS is the most frequent form and is characterized by activating mutations in a number of receptor and transducer molecules, which cause the deliberate activation of the extracellular regulated kinases 1/2 (ERK1/2) and phosphatidylinositol-4,5-bisphosphate 3-kinase (PI3K) signaling pathways [69,71]. Among the oncogenic transducers, RAS is considered a major driver of ERMS etiogenesis [94], as the RAS^G12V^ mutated form is sufficient to convert normal myogenic cell precursors into pre-neoplastic and neoplastic counterparts [82,95]. Accordingly, germline RAS mutations on chromosome 11p15.5 are causative of the Costello syndrome, which predisposes individuals to the formation of embryonic tumors, including ERMS [96]. Sustained RAS activation correlates with ERMS tumor risk and was shown to promote a higher rate of G→T transversions due to high ROS formation [97,98]. As a consequence, RMS tumors were reported to be sensitive to a number of oxidative inducers [97], including auranofin—an inhibitor of thioredoxin reductase [99], cervistatin—a synthetic statin causing mitochondrial impairment [100], and ouabain—a glycoside inhibiting the Na^+^/K^+^ ATPase activity (Table 3). According to these findings, the identification of oxidative-stress inducers represents a milestone for the implementation of the therapeutic regimen of RMS.

### 3.2. Ferroptosis in Rhabdomyosarcoma: State of the Art

Schott et al. found that lentiviral infection of mutated hyperactive RAS forms (NRAS^G12V^, KRAS^G12V^ and HRAS^G12V^) in the RMS13 cell line significantly protected against ferroptosis induced by erastin, RSL3 and auranofin, suggesting that activation of the RAS/ERK pathway may confer protection against oxidative stress [104]. However, experiments recently carried out in our laboratories indicated that RMS cell lines with higher basal RAS/ERK activity are preferentially sensitized towards ferroptosis (manuscript under preparation). Thus, more detailed studies must be carried out to verify the relationship between RAS/ERK signaling, oxidative damage and sensitivity to ferroptosis in RMS. High GSH biosynthesis was reported to be necessary for growth, detoxification, and multidrug resistance in RMS. For example, increased GSH levels and GSH-S-transferase (GSTs) activity were observed in high-grade and metastatic STS treated with doxorubicin [105], as well as in ERMS tumors resistant to doxorubicin, topotecan and vincristine [106]. Moreover, high levels of reduced GSH were found in the serum of RMS patients [107]. These findings suggest that lowering GSH levels could affect RMS survival. Recent studies showed that inhibition of the GST isoenzyme, namely GSH transferase P1-1, with 6-(7-nitro-2,1,3-benzoxadiazol-4-ylthio) hexanol (NBDHEX), potentiates cell death in RMS cells treated with chemotherapeutic agents [102]. In the context of ferroptosis, treatment of RMS with sorafenib, an inhibitor of system Xc^−^ causing the depletion of endogenous GSH, counteracts cell proliferation in vitro and xenograft tumor growth in vivo [103]. However, a Phase 2 trial study did not show consistent effects of sorafenib in RMS tumor cohorts, nonetheless its combination with irinotecan or topotecan is being evaluated in children and young adults with refractory solid tumors [108]. Buthionine-sulfoximine (BSO) is another ferroptosis inducer, which inhibits the first reaction of GSH biosynthesis catalyzed by the γ-glutamylcysteine synthetase (γ-GCSc) [43]. BSO has been shown to be effective in reducing the tumorigenic potential of two rat RMS cell lines in vitro and in vivo [101]. Interestingly, one of the two cell lines used in this study was more resistant to the BSO treatment. Biochemical analysis revealed higher levels of γ-glutamyltranspeptidase (γ-GT) [101], a plasma membrane enzyme of the outer surface responsible for breaking down extracellular GSH to increase cystine absorption [109], ultimately leading to an increase in intracellular GSH levels. Thus, it could be argued that the higher γ-GT levels inhibit ferroptosis by increasing GSH levels. Notably, abnormally high γ-GT enzymatic levels were found in patients with high-grade and metastatic sarcomas [105]. Finally, it has been shown that the treatment of RMS cells with tunicamycin or *N*-glycosidase is sufficient to lower GSH levels and cause cell death, suggesting that protein *N*-glycosylation is required for GSH biosynthesis [110]. In this regard, the folding and auto-catalytic cleavage of γ-GT has been shown to be dependent on *N*-glycosylation [111], suggesting again its potential involvement in ferroptosis resistance. 

## 4. Conclusions

Recent discoveries shed light on a peculiar iron-dependent non-apoptotic form of cell death, namely ferroptosis, the execution of which is dependent on the availability of intracellular free iron, the levels of PUFAs and the levels of enzymes of detoxification from oxidative stress. RMS tumors display hallmarks of oxidative damage, which could predict the susceptibility of RMS to a number of oxidative stress inducers. Despite the important role of iron metabolism and iron proteins in cancer, little work has been done on RMS. Therefore, we discussed the agents involved in ferroptosis activation, believing that a better understanding of this mechanism in RMS may lead to therapeutic improvements.

## Figures and Tables

**Table 1:** Proteins and pathways modulating ferroptosis.

**Pro-Ferroptosis**	**Function**	**References**
ACSL4	Acyl-CoA synthase long-chain 4 increases the fraction of long polyunsaturated ω6 fatty acids in cellular membranes	[55]
CARS	Cysteinyl-tRNA synthetase is an enzyme involved in charging of tRNAs with cysteine for protein translation	[56]
Gln	Glutamine via glutaminolysis is essential for ferroptosis triggered by deprivation of full amino acids or of cystine alone	[57]
HO-1	Heme oxygenase-1 is a heme-degrading enzyme releasing iron	[58]
LOX-5	Lipoxygenase-5 catalyzes the dioxygenation of PUFAs	[8]
NCOA4	Nuclear receptor coactivator 4 promotes H-Ferritin degradation	[27,59]
NOX	NADPH oxidase produces ROS species	[6]
P53	It represses the expression of SLC7A11 encoding a subunit of the system Xc^−^	[60]
SAT1	Spermidine/spermine *N*-acetyltransferase increases the peroxidation of arachidonic acid	[61]
TfR1	Transferrin receptor 1 is involved in the iron uptake	[57]
**Anti-Ferroptosis**	**Function**	**References**
Ferritin	The main intracellular iron storage protein	[62]
GPX4	Glutathione peroxidase-4 is a selenoenzyme neutralizing lipid hydroperoxides	[43]
HSPA5	Heath shock protein-5 prevents GPX4 degradation	[63]
HSPB1	Heat shock protein β-1 protects from lipid ROS	[52]
IRP2	Iron responsive protein-2 controls the transcription of TfR1, Ferritin and FPN	[62]
MT-1	Metallothionein-1 binds heavy metals	[64,65]
Mevalonate pathway	Pathway controlling the biosynthesis of selenoproteins, such as GPX4	[66]
Mitochondrial Ferritin	Iron-storage protein	[67]
NRF2	Nuclear factor erythroid 2-related factor 2 drives a transcriptional antioxidant program	[68]
System Xc^−^	The antiporter involved in cystine absorption	[60]

**Table 2:** Histological classification and molecular drivers of rhabdomyosarcom (RMS).

RMS Histotypes	% of All RMS Cases	Location	Age	Prognosis	Dominant Molecular Drivers
Embryonal	60%	Genitourinary tract, head and neck, urinary bladder, prostate, biliary tract, abdomen, pelvis, retroperitoneum	<10	favorable	Activating mutations in PDGFRA, ERBB2, FGFR4, RAS, PIK3CA [76,77,78,79] IGF-2 overexpression [80,81] Somatic mutations in p53 [82]
Alveolar	20%	Extremities, head and neck, chest, genital organs, abdomen and anal area	10–20	unfavorable	Chromosomal translocation t(2;13)(q35;q14) [83,84] N-MYC overexpression [85] IGF-2 overexpression [81]
Pleomorphic	10%	Extremities, chest and abdomen	60–80	unfavorable	Complex karyotypes with no recurrent structural alterations
Spindle cell	10%	Paratesticular, head and neck	<10 and >40	favorable (children) unfavorable (adults)	NCOA2 gene rearrangements [86] Mutations in MYOD1 [87]

Abbreviations used are: ERBB2, erb-b2 receptor tyrosine kinase 2; FGFR4, fibroblast growth factor receptor 4; IGF-2, insulin-like growth factor 2; MYOD1, myogenic differentiation 1; NCOA2, Nuclear Receptor Coactivator 2; MYC, myelocytomatosis viral related oncogene; PDGFRA, platelet-derived growth factor receptor A; PIK3CA, phosphatidylinositol-4,5-bisphosphate 3-kinase, catalytic subunit alpha; RAS, retrovirus-associated DNA sequences.

**Table 3:** Oxidative stress inducers that have shown efficacy in RMS treatment.

Agents	Targets	Reference
auranofin	Inhibitor of thioredoxin reductase	[97]
buthionine-sulfoximine	Inhibitor of the first step of GSH biosynthesis	[101]
cervistatin	Synthetic statin causing mitochondrial impairment	[97]
NBDHEX	Inhibitor of GSH transferase P1-1	[102]
ouabain	Inhibitor of the Na^+^/K^+^ ATPase activity	[97]
sorafenib	Inhibitor of system Xc^−^	[103]
